# A 36-Year-Old Renal Transplant Recipient Female with Leg Ulcer: A Case Report and Brief Review

**DOI:** 10.1155/2018/5086501

**Published:** 2018-01-11

**Authors:** Ali Monfared, Eftekhari Hojat, Seyed Alireza Mesbah, Abbas Darjani, Seyyede Zeinab Azimi

**Affiliations:** ^1^Department of Nephrology, Urology Research Center, Razi Hospital, School of Medicine, Guilan University of Medical Sciences, Rasht, Iran; ^2^Department of Dermatology, Skin Research Center, Razi Hospital, School of Medicine, Guilan University of Medical Sciences, Rasht, Iran; ^3^Razi Pathobiology Laboratory, Rasht, Iran

## Abstract

**Background:**

Opportunistic infections are common in organ transplant recipients. After 6 months of transplantation, patients have the highest risk of opportunistic infections such as cryptococcosis.

**Case Presentation:**

The report presents the case of a 36-year-old female renal transplant recipient, with complaints of few subcutaneous painful and warm nodules and large, warm, erythematous, nontender plaques on the mildly edematous right leg and ankle. Incisional biopsy of the subcutaneous nodule over the leg showed panniculitis with small- to medium-sized vasculitis associated with round yeast forms, and culture of the fragments revealed *C. neoformans* var. grubii.

**Conclusions:**

This article also reviews in brief the treatment of this rare complication. Reviewing the literature showed that since the cryptococcal cutaneous lesions are often nonspecific, the clinical picture solely is not enough to construct a definite diagnosis and there must be a high clinical suspicion.

## 1. Introduction

Cutaneous disorders in kidney transplant recipients are common. Organ transplant recipients' survival enhanced because of advancements in immunosuppressive therapies. Due to prolonged survival among immunosuppressed patients, it is very important to pay more attention to increased risk of skin lesions and early detection, as well as appropriate management of them [1].

## 2. Case Presentation

**Figure 1:**
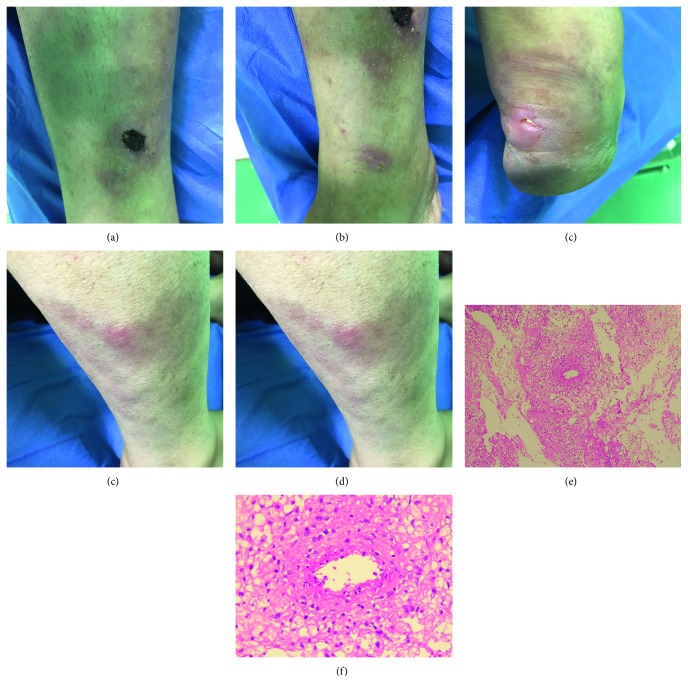
(a to d) Large, warm, erythematous, nontender plaques and subcutaneous nodules on the edematous right leg and ankle in a renal transplant recipient on admission. (e and f) Hematoxylin and eosin shows fat necrosis and coagulative necrosis and rare foci of vasculitis as well as foci of RBC extravasation.

A 36-year-old female, a resident of north of Iran, presented with a complaint of few subcutaneous nodules during a visit to renal transplant clinic. She also had history of large, warm, erythematous, nontender plaques on the mildly edematous right leg and ankle since one month ago. She had been in her usual health status until one month ago. The nodules were painful and warm, and the skin over the nodules consisted of focal superficial ulcerations (Figure 1). She was admitted for further evaluations.

On admission, she was conscious, alert, and oriented. Her vital signs were stable. There was no history of fever, cough, hemoptysis, dysuria, oliguria, headache, or vomiting. Also there was no lymphadenopathy or hepatosplenomegaly. Physical examination by a consultant dermatologist showed multiple erythematous plaques and nodules on the anterolateral portion of right leg and dorsum of calcaneus. There was a central ulceration on one plaque on the leg as well. The margins were discrete. Sanguineous and purulent exudative materials were observed from both margins and center of the lesion (Figure 1). The pulse of the dorsalis pedis artery was well palpable.

She had undergone renal transplantation 15 years earlier for end-stage renal disease due probably to Alport syndrome. Her immunosuppression regimen consisted of prednisolone 5 mg/day, cyclosporine 50 mg/BD, and mycophenolate mofetil 500 mg/BD. Laboratory investigations are shown in Table 1.

The leg ulcer was noted to have signs of infection, and swab cultures were reported as positive for *Escherichia coli*. She was treated with a course of ciprofloxacin and clindamycin, but no improvement was achieved after 96 hr of antimicrobial therapy. She developed erythema, tenderness, and edema of the right calf; the ulcers extended progressively over the tibial crest.

C3 and C4 levels were 93 and 36 mg/dL, respectively; ANCA was negative (3.6) and angiotensin-converting enzyme (ACE) level was 65 *µ*L. Urine analyses and urine culture showed normal results. She was negative for hepatitis B surface antigen, hepatitis B core antibody, hepatitis C virus, human immunodeficiency virus (HIV), and cytomegalovirus (CMV). The purified protein derivative (PPD) test was negative as well. The chest X-ray showed no abnormal lesion.

One day later, an incisional biopsy taken from the subcutaneous nodule over the leg showed panniculitis with small- to medium-sized vasculitis (Figure 1).

**Figure 2:**
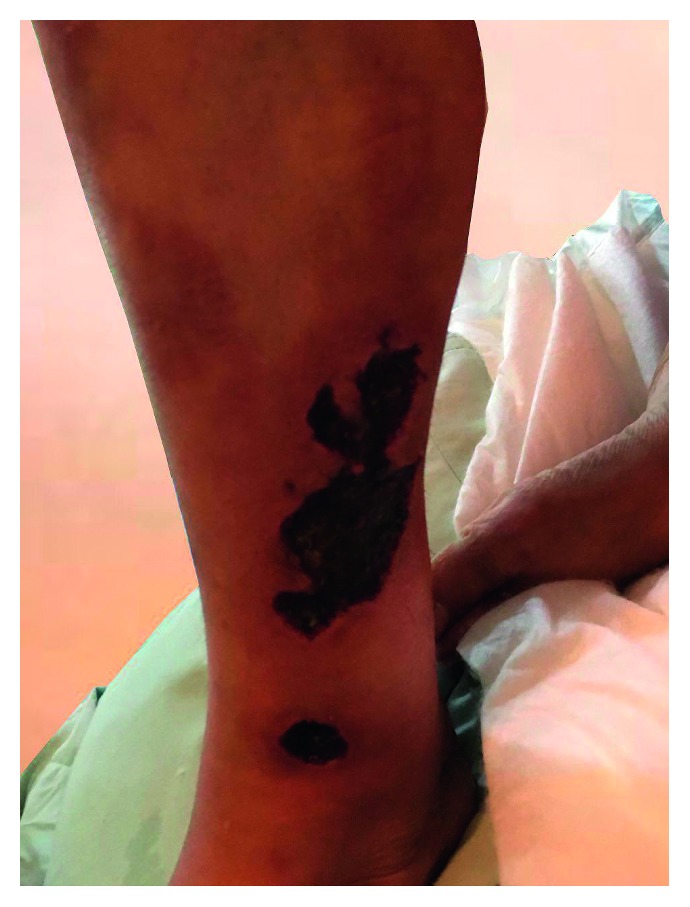
Rapid deterioration of ulcers and increase in the lesion size after 2 weeks.

During this period, lower limb swelling and pain increased. Ulcers developed on her mid to lower leg over the tibial crest and the dorsum of calcaneus, with sharply demarcated red border. The lesions were almost circumferential, with no hyperpigmentation or purpuric-type rash on the skin. There was no peripheral neuropathy. Over a period of a few days, her condition deteriorated, and the ulcerative area rapidly increased in size (Figure 2).

Cyclosporine was changed to rapamune 1 mg/day. Also vancomycine and ceftazidim were prescribed for bacterial super infection. Her renal functions remained stable during therapy.

The culture of tissue biopsy revealed the yeast colonies. Then, she was treated with conventional amphotericin B 50 mg/day and oral fluconazole 200 mg/BD (adjusted due to her renal function) subsequently.

But, the patient developed edema and tenderness over the right leg, with extensive ulcerations of her right leg and foot. The result of a test received. Results of direct India ink examination and culture of an aspirate from the leg were positive for cryptococcus.

**Figure 3:**
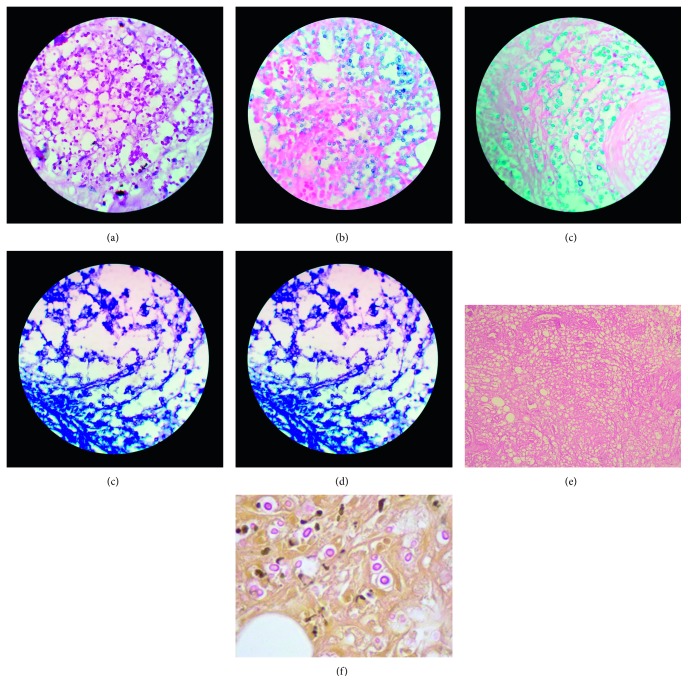
(a) Periodic acid-Schiff (PAS), (b) alcian blue, and (c) colloidal iron stains were positive, but (d) Giemsa and (e) Prussian blue stains were negative for cryptococcus. (f) Mucicarmine stain was positive for cryptococcosis.

Reevaluation of skin biopsies showed the presence of chronic panniculitis associated with round yeast forms. Unless specifically searched for, the organisms were difficult to find on hematoxylin and eosin preparations. Periodic acid-Schiff reagent, alcian blue, and mucicarmine showed single, small yeast forms surrounded by a clear halo. These organisms were present in moderate numbers in the fat and occasionally in the reticular dermis. Giemsa and Prussian blue stains were negative (Figure 3). Foci of fat necrosis, coagulative necrosis, and rare foci of vasculitis involving small- to medium-sized vessels resulting luminal occlusion were also evident, as well as foci of RBC extravasation. The above findings were suggestive of cryptococcal panniculitis associated with secondary vasculitis.

The culture of biopsy from the leg's necrotic ulcer grew *C. neoformans* var. grubii. The overall colonies appearance was creamy, smooth, and mucoid.

The serum cryptococcal antibody titer was positive (1 : 16), and it was confirmed by polymerase chain reaction (PCR). Cerebrospinal fluid was negative for cryptococcus. No evidence of disseminated disease was found. Two weeks later, she developed right knee arthritis which was negative for cryptococcus in synovial assay. Blood cultures were also negative. Amphotericin B was continued (1 mg/kg/day). Chest radiography was normal. Subsequent questioning revealed that she had no contact with pigeons.

The skin lesions regressed gradually, and ulcers started healing, however leaving eschars. Black eschars remained persistent despite administration of fibrinolysin ointment. Surgical debridements were carried out to remove the necrotic tissue, and then graft was performed for her. The plan is to continue fluconazole for at least 1 year.

## 3. Discussion

Cutaneous disorders in kidney transplant recipients are common. Organ transplant recipients' survival enhanced because of advancements in immunosuppressive therapies. Due to prolonged survival among immunosuppressed patients, it is very important to pay more attention to increased risk of skin lesions and early detection, as well as appropriate management of them. These skin conditions include aesthetic distortion, iatrogenic lesions due to immunosuppressive drugs, infections, precancerous lesions, and malignancies [1].

Opportunistic infections are common among organ transplant recipients [1]. Cutaneous manifestations of infection in immunosuppressive recipients may be an important clue to their existence [2, 3].

Skin infections may be primary or secondary. Secondary cutaneous infections are because of an underlying systemic or disseminated infection. In the first postoperative month, most infections are due to nosocomial infections and surgical complications [4]. *Staphylococcus* and *Streptococcus* superficial pyodermas, cellulitis, and wound infections are the most common infections during this period [1]. Moreover, atypical pathogens contribute to soft tissue and wound infections in this group. Reactivation of herpes simplex virus (HSV) often occurs within the first 3 postoperative weeks. The most common presentation of HSV is anogenital and orolabial lesions [5].

In 2 to 6 months after renal transplantation, opportunistic infections with organisms such as atypical mycobacterial species, mycobacterium tuberculosis, CMV, Epstein-Barr virus, nocardia, and bartonella are common [4, 6].

After 6 months of transplantation, patients with inadequate graft function need higher than usual immunosuppressive dosage. So these patients have the highest risk of opportunistic infections such as *Pneumocystis carinii*, cryptococcosis, and nocardiosis as well as reactivation of varicella-zoster virus [7].

The skill to connect particular pathogens and infections with compromised immunity can help to set up a differential diagnosis and direct a logical management plan. The convenience of the skin to rapid diagnostic sampling procedures can save lives and prevent the most invasive kinds.

Fungal infection following solid-organ transplantation is a major cause of morbidity and mortality. After candidiasis and aspergillosis, cryptococcosis is the third most common invasive fungal infection in organ transplant recipients. The immunosuppressive regimens and antirejection therapy with pulse-dose methylprednisolone used in solid organ transplantation might have been considered as the predisposing factor [8, 9].

*Cryptococcus neoformans* is present in soils enriched by bird excreta; fruits may also harbor the yeast. It is usually acquired through inhalation of airborne spores [9, 10]. However, our patient had no history of cutaneous injury, outdoor activities, and exposure to bird droppings. In the past few years, it became evident that direct inoculation of the pathogen through a minor skin wound can cause primary infection of the skin [11].

In immunocompetent hosts, the infection is usually limited to the lungs, with self-limited flu-like symptoms. Although cryptococcosis has been commonly encountered among the HIV-infected population, there are reports of cryptococcosis in non-HIV-infected patients such as human stem cell transplant (HSCT) recipients, solid organ transplant recipients, patients with hematologic malignancies, and patients with other malignancies [10].

Cutaneous cryptococcosis is a manifestation of disseminated infection, and therefore, it should prompt a comprehensive investigation into cerebrospinal fluid, blood, the lungs, and serum cryptococcal antigen. Usually, cryptococcosis starts as a respiratory tract infection, followed by hematogenous dissemination to other organs. Primary cutaneous cryptococcosis is defined as lesions confined to a restricted body region with positive skin cultures for *C. neoformans* and without signs of concurrent dissemination [12].

A few cases of cutaneous lesions with no evidence of systemic involvement have been reported, but this makes controversial with current knowledge that cutaneous involvement points to disseminated disease [13].

About 15% of patients with cryptococcosis have skin lesions. The most common cutaneous presentations of cryptococcosis are papules, nodules on the skin resembling molluscum contagiosum, vesicles, nodules, mass, pustule, abscess, ulcers, ecchymosis, and rarely cellulitis. Necrotizing fasciitis, eschar, ecchymosis, panniculitis, vegetating plaques, palpable purpura, and pyoderma gangrenosum-like lesion and cellulitis with necrotizing vasculitis have also been reported in the organ transplant recipients [12, 13].

In transplant recipients, drug-induced immune deficiency results in the extrapulmonary hematogenous spreading of cryptococcosis, with the involvement of other organs, such as the central nervous system (CNS), as well as the cutaneous tissue, urinary system, bones, and joints. Cutaneous lesions may be the only sign of severe systemic disease in transplant patients. Not only do cutaneous lesions largely characterize hematogenous dissemination, but the skin also has been recognized as a portal of access to cryptococcus and a potential source of following disseminated disease in transplant recipients [10].

Since the cryptococcal cutaneous lesions are often nonspecific, the clinical picture solely is not enough to construct a definite diagnosis and there must be a high clinical suspicion. We need deep skin biopsy consisting of soft tissue to make the diagnosis of panniculitis. With only scraping the skin or aspiration material, one may identify fungal infection, but panniculitis would be missed. This may elucidate why only occasional case reports have revealed fungal panniculitis in posttransplant patients [9].

Only 14 cases of cryptococcal panniculitis in solid organ transplant recipient have been reported so far. After reviewing all these cases of cryptococcal panniculitis, it has been estimated that duration between organ transplant and cryptococcal infection ranges from 3 months to 31 years with a mean period of 9.64 years. Age of onset of cryptococcal infection varies from 33 to 61 years, and the mean age of onset of panniculitis was 49.64 years [10].

## 4. Management

In immunocompromised patients, we need to evaluate systemic involvement in a complete assessment including a collection of large volume CSF (≥1 ml or 20 drops) and blood and urine analysis (10). About 88–91% of the organ transplant recipients with cryptococcal meningitis show positive serum cryptococcal antigen; notably in non-HIV-infected recipients, the serum and CSF antigen titers are usually lower. Localized cutaneous lesion in HIV-infected patients that did not develop AIDS is often antigen negative [14].

The Infectious Diseases Society of America (IDSA) updated guidelines on the management of cryptococcal disease consist of categories based on the organ involvement. Patients with central nervous system (CNS) disease should be treated with liposomal amphotericin B (AmB) (3-4 mg/kg/day intravenously) or amphotericin B lipid complex (5 mg/kg/day intravenously) plus flucytosine (100 mg/kg/day in four divided doses) for at least two weeks, followed by oral fluconazole (400 to 800 mg (6 to 12 mg/kg) per day) for eight weeks and then followed by a lower dose of oral fluconazole (200 to 400 mg/day) for six to 12 months.

If flucytosine was not prescribed from the beginning, four to six weeks of therapy with liposomal formulations of amphotericin B should be considered. Patients with a high-fungal burden or those on relapse benefit from liposomal amphotericin B (6 mg/kg/day).

In immunocompetent patients, disseminated, non-CNS cryptococcus infection is treated with oral fluconazole for 3–6 months or with itraconazole for 6–12 months.

In immunosuppressed patients, the initial treatments are the same, but lifelong maintenance treatment with fluconazole may be required. The primary cutaneous disease can be treated with oral fluconazole or itraconazole [15].

The present case was also started with conventional amphotericin B 50 mg/day without flucytosine in the induction phase of therapy and was followed by fluconazole 200 mg/twice a day. Our patient showed significant clinical improvement after systemic antifungal therapy.

## Figures and Tables

**Table 1:** Laboratory data on admission and one month later.

Laboratory data	Normal range adults	On admission	One month after admission
White cell count (per mm^3^)	4–10	9.5	7.2
Differential count (%)			
Neutrophils	40–60	85	83
Lymphocytes	20–40	13	10
Monocytes	4–8	2	2
Eosinophils	1–3	—	1
Basophils	0–1	—	1
Band forms	0–5	—	—
Red cell count (per *µ*L)	4.1–5.1	4.7	3.3
Mean corpuscular volume (fl)	80–100	80.6	81.3
Mean corpuscular hemoglobin (pg/red/cell)	27–32	24.6	26.1
Mean corpuscular hemoglobin concentration (g/dL)	31–37	29	31.3
Red-cell distribution width (%)	10.6–15.7	13.8	14.5
Hemoglobin (g/dL)	14–18	9.7	9
Hematocrit (%)	42–52	40.03	30
Platelet count (per mm^3^)	150–450	313	189
Sodium (mmol/liter)	135–145	139	135
Potassium (mmol/liter)	3.5–5.3	3.8	3.7
Calcium (mg/dL)	8.5–10.5	8.1	7.5
Phosphorus (mg/dL)	2–4	3.5	3.2
Blood urea nitrogen (mg/dL)	7–21	41	37
Creatinine (mg/dL)	0.6–1.1	2.5	1.5
Estimated glomerular filtration rate (ml/min/1.73 m^2^)	90	27.01	45.01
Glucose (mg/dL)	140	145	125
Total protein (g/dL)	6.6–8.8	6.1	5.9
Albumin (g/dL)	3.5–5	3.6	3.8
Alanine aminotransferase (U/liter)	<37	45	40
Aspartate aminotransferase (U/liter)	<41	36	37
Alkaline phosphatase (U/liter)	80–306	93	100
Total bilirubin (mg/dL)	0–1.1	0.7	0.6
Direct bilirubin (mg/dL)	0–0.2	0.2	0.2
Iron (*µ*g/dL)	30–150	55	7
Iron-binding capacity (*µ*g/dL)	230–400	216	230
Ferritin (ng/ml)	10–100	54	55
